# The association between interleukin-28B gene polymorphisms as a potential biomarker and the risk of chronic Periodontitis in an Iranian population

**DOI:** 10.1186/s13005-017-0148-y

**Published:** 2017-06-27

**Authors:** Zahra Heidari, Bita Moudi, Hamidreza Mahmoudzadeh-Sagheb, Mehrnoosh Moudi

**Affiliations:** 1 Infectious Diseases and Tropical Medicine Research Center, Zahedan University of Medical Sciences, Zahedan, Iran; 2 Department of Histology, School of Medicine, Zahedan University of Medical Sciences, Zahedan, Iran; 3 Department of Biology, Science and Research Branch, Islamic Azad University, Tehran, Iran

**Keywords:** Chronic Periodontitis (CP), Interleukin-28B (IL-28B), Polymorphism

## Abstract

**Background:**

Chronic Periodontitis (CP) is a common inflammatory disease affects supporting tissues of the teeth and can lead to tooth loss. The objective of this study was to determine the relationship between polymorphisms in the *IL-28B* gene and chronic periodontitis in an Iranian population.

**Methods:**

Two hundred and ten CP patients and one hundred healthy subjects were enrolled in the present case-control study. The *rs12979860* and *rs8099917* SNPs were identified using RFLP and T-ARMS-PCR methods respectively.

**Results:**

SNP analysis revealed that the G allele of *rs8099917* SNP and T allele of *rs12979860* SNP increased susceptibility to CP compared to the A allele and C allele (*p* < 0.0001, OR = 2.712, CI = 1.783-4.126; *p* < 0.0001, OR = 2.538, CI = 1.784-3.613 respectively). In addition, the CT/GT, TT/GG and TT/GT haplotypes were predominant in CP patients and significantly associated with the increased risk of CP.

**Conclusion:**

*IL-28B* polymorphisms may be useful predictive factors for chronic periodontitis and correlated to the susceptibility to CP infection in our population.

## Background

Chronic Periodontitis (CP) is one of the most common inflammatory diseases affecting the tooth supporting tissues. This Chronic condition is caused by microorganisms that produce dental biofilm on the tooth surfaces. Bacterial plaque triggers the initiation of the inflammation and induces immune responses against infection in the host body. CP destroys soft tissue and the bone that supports the teeth and eventually leads to tooth loss and other serious conditions such as heart attack in progressive modes. CP is an important cause of tooth loss in 10–15% of adults [1]. In regard to the etiology of CP, it seems that bacteria, immune interactions, environmental and genetic factors be responsible [1]. It has been demonstrated that CP related inflammation can affect host immune system by stimulations on the level of cytokines production [2]. On the other hand, twin studies have revealed that genetic factors can modulate the expression of immune mediators and increase the risk of CP [3]. Cytokines and chemokines; as immune mediators, have important roles in pathogenesis of CP [4, 5]. Interleukin-28 (IL-28), also known as IFN-λ, have important role in immune responses against infections. It can modulate the innate and adaptive immune systems against chronic inflammations [6]. This cytokine has two isoforms: IL-28A and IL-28B [7]. In the human genome, interleukin-28A (IL-28A), IL-28B and IL-29, also known as interferon-λ1 (IFN-λ1), IFN-λ2 and IFN-λ3 respectively, have produced a cluster of closely related genes. These genes have biological functions and antiviral activity and can be induced by various infections [6–10].

Sanders et al. [11], reported the first GWAS of CP among a large community-based sample of Hispanics/Latinos (10,935 adult participants). Genotyping was done with approximately 20 million single-nucleotide polymorphisms. They identified a genome-wide significant association signal in the 1q42.2 locus and four more loci with suggestive evidence of association in 1q22, 5p15.33, 6p22.3 and 11p15.1.

Lopes et al. [12], observed a significantly increased risk of developing chronic periodontitis in individuals with low IL-10 production. They suggested that the polymorphisms A-1082G, C-819 T, and C-592A, are involved in the susceptibility to the development of chronic periodontitis in an admixed northern Brazilian population.

Zhu et al. [13], in an updated meta-analysis of 21 case-control studies, reported that IL-6174 polymorphism is associated with CP susceptibility. Also they revealed that IL-6174 GG genotype plays a role as a risk factor to CP in Brazilian and Caucasian population.

In another meta-analysis, Yang et al. [14], investigated the association between the IL-8 -251A/T polymorphism and the risk of periodontitis. The results suggested that the IL-8 -251A/T polymorphism may increase the risk of periodontitis in Asian and mixed populations.

Lavu et al. [15], in the study of clinical relevance of cytokines gene polymorphisms and protein levels in gingival cervical fluid from chronic periodontitis patients, revealed the presence of higher levels of IL-1β and TNF-α in subjects with periodontitis and genetic control of IL-1β levels in Indians.

Recently, Sheibak [16] et al., studied the quantitative parameters of interdental gingiva in chronic periodontitis patients with IFN-γ gene polymorphism. They found that IFN-γ +874 A/T is strongly associated with some quantitative parameters of connective tissue constituents of interdental papilla in CP patients.

Heidari et al. [17] investigated the association of IFNL3 gene polymorphisms (rs12979860 and rs8099917) with HBV susceptibility, in chronic HBV-infected patients. Their study showed no significant differences between patients, with at least one rs12979860C and or rs8099917T alleles compared to the healthy controls.

The gene encoding the IL-28B cytokine is located on the long arm of chromosome 19 at position 19q13.13 [18]. Recently, several studies have revealed that genetic variations such as single nucleotide polymorphisms (SNPs) at or near the *IL-28B* gene can affect the natural history of chronic infections [19–21].

In addition, it has been shown that the expression levels of the IL-28B receptor mRNA increase in antiviral protection in some human organs such as thyroid and pancreas [6, 8]. Recently, Cheng et al. [22] and Osaki et al. [23] have reported that IL-28B gene polymorphisms can affect the development of hepatitis B virus infection. Based on previous studies concerning the effects of this gene SNPs on inflammatory diseases, it seems that *interleukin-28B* gene variations might have a vital role in the susceptibility to CP. Two SNPs have been found in the *IL-28B* gene at positions *rs8099917* and *rs12979860* which associated with the response of individuals to chronic infections [24]. In our knowledge, therefore we assumed that these variations might be possible markers for the detection of CP and the current study was the first investigation that has been conducted on this issue.

Previously, we have studied the relationship between polymorphisms of inflammatory and proinflammatory genes and chronic periodontitis [4, 25–30]. In this study, given the pervasiveness of chronic periodontitis in Iran and the importance of *IL-28* in the pathogenesis of inflammation, we decided to examine the impact of these cytokine SNPs in susceptibility to CP.The aim of this paper is to investigate the association between two single nucleotide polymorphisms, *rs8099917* and *rs12979860*, and CP.

## Methods

### Study subjects

This case-control study was done on 210 CP patients and 100 healthy individuals who were referred from September 2015 until March 2016. All subjects were exclusively Iranian ethnicities from the region of Sistan and Baluchistan. Patients with chronic periodontitis were examined at the Periodontology Department, Dentistry Clinic of Zahedan University of Medical Sciences (ZUMS).

The study was approved by the Institutional Ethics Committee of the Zahedan University of Medical Sciences (No: 6210) and written consent forms were signed by all participants. The study was carried out in Infectious Diseases and Tropical Medicine Research Center, Zahedan, Iran. Chronic periodontitis patients were diagnosed based on the criteria of the International workshop for classification of periodontal diseases and conditions [31]. The disease diagnosis was based on physical examination, medical and dental history, probing depth (measured as the distance from the gingival margin to the bottom of the pocket), and assessment of clinical attachment loss (as the distance from the cement-enamel junction to the bottom of the periodontal pocket). Probing was performed at six sites around each tooth using a WHO periodontal probe and recording the maximum values, tooth mobility, and radiographs. The loss of alveolar bone was determined radiographically [26, 32]. All participants were nonsmokers, had at least 20 teeth and were Iranian ethnicities from the South East of Iran and were of good general health. Clinical evidence in healthy subjects included: GI < 1 and PPD < 3 mm, and CAL = 0 and they had a healthy periodontium. Signs of clinical inflammation such as GI > 1, PPD > 4 mm, and CAL > 2 mm and bone loss were clinical evidence for chronic periodontitis. Patients were excluded from the study if they had a history of cardiovascular disorders, systemic disorders, immunodeficiency situations and conditions such as use of anti-inflammatory drugs, chemotherapy, individuals with previous orthodontic treatment, pregnant women and smokers.

The control group consisted of 100 unrelated healthy individuals who had no clinical history of periodontal disease. Controls were selected from subjects referred to the Dentistry Clinic for reasons other than periodontal disease and were matched for age, ethnicity, and gender with CP group. There are three main ethnicities in South-East of Iran; Baluch, Sistani and others.

### Genotyping of IL-28B polymorphisms (*rs8099917* and *rs12979860*)

Two ml peripheral blood was taken from all participants in Na-EDTA tubes. Genomic DNA was isolated from peripheral venous blood using salting-out method as described previously [33]. Allele Specific primers were designed using the Primer BLAST tool from NCBI (https://www.ncbi.nlm.nih.gov/tools/primer-blast/) as shown in Table 1.

**Table 1:** The primer’s sequences used for detection of IL-28B (*rs8099917* and *rs12979860*) gene polymorphisms using RFLP-PCR and T-ARMS-PCR

Polymorphisms	Primer Sequence (5’- > 3’)
*rs12979860*	Forward: 5’-GCTTATCGCATACGGCTAGG-3’
	Reverse: 5’- AGGCTCAGGGTCAATCACAG −3’
*rs8099917*	Forward outer: 5’-CATCACCTATAACTTCACCATCCTCCTC-3’
	Reverse outer: 5’-GGTATCAACCCCACCTCAAATTATCCTA-3’
	Forward inner[G allele]: 5’-CTTTTGTTTTCCTTTCTGTGAGCAGTG-3’
	Reverse inner [T allele]: 5’-TATACAGCATGGTTCCAATTTGGGTAAA-3’

**Fig. 1:**
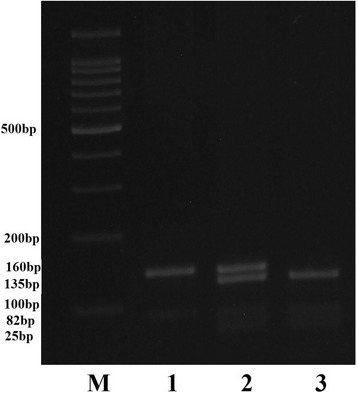
Electrophoresis pattern of PCR-RFLP for detection of *IFNL3* rs12979860 C/T polymorphism. Lane M, DNA marker; lane 1, subjects with homozygote TT genotype; lane 2, subjects with heterozygote CT genotype; lane 3, subjects with homozygote CC genotype

The *rs12979860* polymorphism was identified using polymerase chain reaction (PCR) based restriction fragment length polymorphism (RFLP) assay. A 242 base pair (bp) product was obtained. PCR amplification was carried out in a total volume of 20 μL containing 1 μl of each primer, 100 ng of template DNA and 10 μl of 2X Prime Taq Premix (Genet Bio, Korea) and 7 μl ddH2O. Samples were subjected to 35 cycles of initial denaturation; 5 min at 95 °C, denaturation; 30 s at 95 °C, annealing; 30 s at 60 °C, extension; 30 s at 72 °C, final extension; 5 min at 72 °C. 10 μl of the amplicons were digested with 1 unit of the BstU-I restriction enzyme (Fermentas, Vilnius, Lithuania) at 60 °C overnight. IL-28B-C allele gives 3 fragments of 135 + 82 + 25 bp, and IL-28B-T allele 2 fragments of 160 + 82 bp. The fragments were resolved by electrophoresis in 4% agarose gel after staining with ethidium bromide (Fig. 1.).

**Fig. 2:**
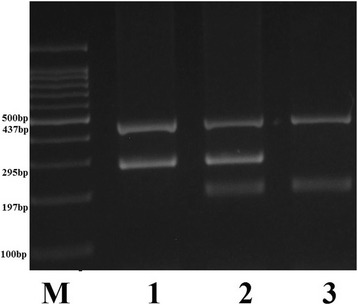
Electrophoresis pattern of ARMS-PCR for detection of *IFNL3* rs8099917 G/T polymorphism. Lane M, DNA marker; lane 1, subjects with homozygote TT genotype; lane 2, subjects with heterozygote GT genotype; lane 3, subjects with homozygote GG genotype

The *rs8099917* polymorphism was determined using tetra-primer amplification refractory mutation system–polymerase chain reaction (T-ARMS-PCR) method as described previously [34]. Amplification was performed in a volume of 25 μL containing 1 μl (10 μM) of each primer, 100 ng of template DNA and 10 μl of 2X Prime Taq Premix and 10 μl ddH2O. Polymerase chain reactions were run for 30 cycles: initial denaturation; 5 min at 95 °C, denaturation; 30 s at 95 °C, annealing; 30 s at 58 °C, extension; 30 s at 72 °C, final extension; 10 min at 72 °C. Product sizes were 197 bp for G allele, 295 bp for T allele, and 437 bp for the two outer primers (control band). Each reaction was verified on a 2% agarose gel containing ethidium bromide (Fig. 2.).

### Statistical analysis

All statistical analysis was performed using SPSS version 20.0 software. The Chi-square test was used to assess the descriptive statistics. A *P* value less than 0.05 was considered statistically significant. The genotypic and allelic frequencies observed in patient and control groups were calculated by direct counting. The associations between the allelic and genotype frequencies and CP, as well as the odds ratio (OR) for the susceptibility to disease were obtained by the χ2-test and 95% confidence intervals (95% CI) from logistic regression analyses. Quantitative data were presented as mean ± standard deviation.

## Results

The study population was composed of 210 chronic periodontitis patients (mean age 28.33 ± 5.765; 95 female and 115 male) and 100 healthy controls (mean age 29.22 ± 3.597; 52 female and 48 male). The clinical data showed that the values of the gingival index (GI), probing pocket depth (PPD), and clinical attachment level (CAL) in CP group were higher than healthy controls (*p* < 0.05). The demographic data showed that the mean age, ethnicity and gender for patients with CP and healthy subjects did not differ between the two groups (*p* = 0.159, *p* = 0.186, *p* = 0.265 respectively) [26].

The allele frequencies and genotype distributions for the two interleukin-28B gene polymorphisms (*rs8099917* and *rs12979860*) among CP patients and control group were shown in Table 2. The genotype frequencies were in agreement with the Hardy–Weinberg equilibrium. The frequencies of *IL-28B rs8099917* and *rs12979860* genotypes in the chronic periodontitis population were significantly different from the healthy group (*p* < 0.0001). It was revealed that the GG (OR = 5.00; CI = 1.656-15.096) and GT (OR = 3.269; CI = 1.915-5.581) genotypes from *rs8099917* SNP and TT (OR = 6.487; CI = 3.001-14.025) and CT (OR = 4.478; CI = 2.529-7.927) genotypes from *rs12979860* SNP were significantly related to the increased risk of CP. In addition, our results indicated significant differences in the distribution of alleles between the two groups at the *rs8099917* and *rs12979860* gene polymorphisms (Table 3). SNP analysis for *rs8099917* and *rs12979860* SNPs revealed that the G allele and T alleles increased susceptibility to CP compared to the A allele and C allele (*p* < 0.0001, OR = 2.712, CI = 1.783-4.126; *p* < 0.0001, OR = 2.538, CI = 1.784-3.613 respectively). The frequencies of the haplotypes in *IL-28B* gene in CP patients and controls depicted in Table 3. There was a significant difference in the haplotype frequencies between chronic periodontitis patients and controls (*p* < 0.0001). The CT/GT, TT/GG and TT/GT haplotypes were predominant in CP patients and significantly associated with the increased risk of CP.

**Table 2:** The frequency of genotypes and alleles of *IL-28B* (*rs12979860* and *rs8099917*) polymorphism gene

IL-28B polymorphisms	CP, N. (%)	Control, N. (%)	OR (95%CI)	P
*rs12979860C/T*				
CC	32 (15.2)	47 (47.0)	Ref = 1	-
CT	125 (59.5)	41 (41.0)	4.478(CI = 2.529-7.927)	0.000
TT	53 (25.2)	12 (12.0)	6.487(CI = 3.001-14.024)	0.000
CT + TT	178 (48.8)	53 (53.0)	4.933(CI = 2.863-8.498)	0.000
Allele				
C	189 (45.0)	135 (67.5)	Ref = 1	-
T	231 (55.0)	65 (32.5)	2.538(CI = 1.784-3.613)	0.000
*rs8099917G/T*				
GG	24 (11.4)	4 (4.0)	5.000(CI = 1.656-15.096)	0.004
GT	102 (48.6)	26 (26.0)	3.269(CI = 1.915-5.581)	0.000
TT	84 (40.0)	70 (70.0)	Ref = 1	-
GT + GG	126 (60.0)	30 (30.0)	3.500(CI = 2.104-5.823)	0.000
Allele				
G	150 (35.7)	34 (17.0)	2.712(CI = 1.783-4.126)	0.000
T	270 (64.3)	166 (83.0)	Ref = 1	-

**Table 3:** Haplotype frequencies in chronic periodontitis (case) and normal subjects (control)

Haplotypes	CP group N (%)	Control group N (%)	P.value	Odds Ratio
CC/GT	14 (6.7)	17 (17)	REF = 1	-
CC/TT	22 (10.5)	32 (32)	0.691	0.835(CI = 0.342-2.036)
CT/GG	10 (4.8)	3 (3)	0.063	4.048(CI = 0.929-17.629)
CT/GT	63 (30)	8 (8)	0.000	9.562(CI = 3.446-26.533)
CT/TT	48 (22.9)	28 (28)	0.090	2.082(CI = 0.892-4.856)
TT/GG	14 (6.7)	1 (1)	0.010	17.000(CI = 1.983-145.729)
TT/GT	25 (11.9)	1 (1)	0.002	30.357(CI = 3.643-252.974)
TT/TT	14 (6.7)	10 (10)	0.334	1.700(CI = 0.579-4.989)
TOTAL	210 (100.0)	100 (100.0)		

## Discussion

According to our knowledge, the current investigation is the first study conducted in any population regarding to the association between *IL-28B* single nucleotide polymorphisms (*rs8099917* and *rs12979860*) and chronic periodontitis. This study showed a higher frequency of *rs12979860* CT + TT and *rs8099917* GT + GG genotypes in CP patients compared to healthy subjects. Our findings revealed that T allele of *rs12979860* and G allele of *rs8099917* were the predominant alleles among CP patients in comparison to healthy controls. Our study demonstrated the *rs8099917* and *rs12979860* SNPs are probably genetic risk factors for susceptibility to CP.

IFN- λ family has been divided into three subtypes: interferon λ1, λ2 and λ3. Interleukin-28B or interferon-λ3 is an endogenous antiviral cytokine which is required to control the chronic infections. Recently, it was revealed that IFN-λ could inhibit the viral replication in human cells such as hepatocytes [6]. The IL-28B cytokine induces signal transduction through the heterodimer receptor in the cells. Complex of the IL-28B and its receptor can activate the immune mediator transcription and Janus kinase-signal transducer. In addition, IL-28B is related to IFN-α that controls above mentioned mechanisms which leads to inhibition of cell proliferation, and regulation of immune functions [10, 35]. Therefore, it seems that variation of IL-28B can lead to changes in the regulation of immune activity. On the other hand, IL-28B have important roles in various organs such as heart, thyroid, pancreas and skeletal muscle because high levels of IL-28B receptor mRNA expression has been shown in these organs when an antiviral protection induced [6]. Also, IL-28B transmits information via IL-10 signal cascade. The amount of IL-10 cytokine increases in various inflammations [36]. These facts reveal an association between IL-28B and chronic periodontitis as an inflammatory infectious disease.

On the other hand, it has been shown that IFN-λ polymorphisms were associated to chronic infection outcome and could affect the progression of inflammations [37, 38]. Studies have reported that *IL28B rs129798060* was related to the susceptibility to chronic hepatitis C virus (HCV) infection. HCV infected patients with the CC genotype of *rs129798060* were presented convincing response to antiviral therapies [19, 20, 39] and also T allele has been reported as a risk factor for HCV infection [40, 41]. It has been confirmed that the *rs129798060* polymorphism could increase the expression of IL-28B [19]. Our findings are in agreement with previous studies [19, 20, 39]. The frequency of *IL28B* CT + TT genotypes in chronic periodontitis patients was higher than in controls. It means that T allele is a risk factor for CP. In addition, subjects with CC genotype may be protected against CP inflammation. Moreover, it was revealed that *rs12979860* and *rs8099917* had vital roles in infection treatment [20, 39]. The IL-28B cytokine can modulate immune responses and change the natural history of other infections but the function of *IL-28B* polymorphisms in our recently study presents that *rs12979860* CT + TT and *rs8099917* GT + GG genotypes are not associated with susceptibility to hepatitis B infection [42]. It seems that IL-28B can regulate HCV related- liver infection [43], so these facts suggested that *IL-28B* gene polymorphisms such as SNPs might have important roles in development of CP as an inflammatory disease.

Recently, Xiao et al. [21], Rauch et al. [44], Derbala et al. [45], Lampertico et al. [46], Egli et al. [47], Eurich et al. [48] have reported that *IL-28B* polymorphisms associate with the susceptibility to the hepatitis B or C viruses infections, influenza and lower urinary tract symptoms (LUTS). Xiao et al. [21] determined the relationship between *IL-28B rs12979860* and *rs8099917* SNPs and LUTS in Chinese patients. In the *IL-28B rs12979860* and *IL-28Rα rs10903035* also *IL-28B rs8099917* and *IL-28Rα rs10903035* interactions analysis, they found that the CC + AG/GG, CT + AG/GG; and TT + AG/GG, GT + AG/GG genotypes were significantly less frequent in the patients compared to the controls. Rauch et al. [44] reported that the *rs8099917* minor allele was associated with progression to chronic infection. The *rs8099917* was also associated with failure to respond to therapy in patients with HCV genotype 1 or 4. Derbala et al. [45] indicated that the CC and TT genotypes of *rs12979860* and *rs8099917* were the more common protective genotypes among infected patients. Egli et al. [47] have identified IL-28B as a key regulator of the Th1/Th2 balance during influenza vaccination. They have demonstrated that the *IL-28B* TG + GG genotypes of *rs8099917* were associated with increased seroconversion following influenza vaccination. Eurich et al. [48] have examined the role of *rs12979860* SNP in the development of hepatocellular carcinoma (HCC). They showed that the prevalence of HCC in explanted livers was significantly higher among patients with TT genotype, suggesting a protective role of the C allele in HCC development. In this field, T allele may be regarded as a genetic risk factor for HCV-related severity. Also, as Rizk et al. [49], reported the patients with the C allele of *rs12979860* exhibited an approximately eight times higher risk of disease severity compared to patients with the T allele. However, chronic periodontitis is still poor understood and the relationship between *rs12979860* and *rs8099917* and CP has not been reported till now but our results showed that these SNPs were associated with susceptibility to CP. Our finding revealed that most frequently genotypes in CP patients were CT + TT for *rs12979860* and GT + GG for *rs8099917* which differs from the study of Barreiro et al. [43] and Rizk et al. [49] but in agreement with many studies by Xiao et al. [21], Derbala et al. [45], Egli et al. [47], Eurich et al. [48].

Due to the above mentioned studies and current investigation, it might be presented: that *rs12979860* T allele and *rs8099917* G allele might be related to the development of chronic infections such as CP, HBV, HCV, LUTS and influenza. Data shows that the C/T alleles and CC/TT genotypes for *rs12979860* and *rs8099917* respectively occurred frequently in the healthy control group and suggest that the *rs12979860* C allele and *rs8099917* T allele may useful for inhibition of chronic periodontitis infection and have a protective effect for inflammation. We considered that, a number of differences between current report and Barreiro et al. [43] and Rizk et al. [49] findings might result from different ethnicities of the study populations.

Together with the significant differences of the distributions of the rs12979860 C/T and rs8099917 G/T alleles in the CP and healthy subjects, our findings seemed to propose that the *IL-28B* SNP might be associated with the risk of CP. In addition, with regards to the stereological analyzes according to the volume of pulp, epithelium, connective tissue, collagenous and non-collagenous matrix, and blood vessels between control and CP groups, it was presented that the *transforming growth factor-β1 29C/T* [30] and *-509C/T* [29], *tumor necrosis factor-alpha -308G/A* [27, 33], *interleukine-6-174G/C* [4] gene polymorphisms were associated with level of tissue breakdown and periodontal disease progression. These studies supported our conclusion that *IL-28B* SNPs have an influence on the natural history of chronic periodontitis in which that the subjects who carried the *rs12979860* CT/TT or *rs8099917* GT/GG genotypes display increased clinical severities of CP than individuals carrying *rs12979860* CC or *rs8099917* TT variants. It means that *rs12979860* C and *rs8099917* T alleles may be protective factors in the field of the development of CP. When the haplotype effects of *rs12979860* and *rs8099917* were analyzed, it was found that CT/GT, TT/GG and TT/GT genotypes have more frequent in CP patients that support our conclusion. One of our hypotheses is that *IL-28B* SNPs can change the expression and secretion of proinflammatory cytokines in patients with CP and finally affect the development of CP. However, the real functional mechanism of *IL-28B* SNPs in the development of CP is still unknown. In addition, there are a number of limitations in our study. It seems that chronic infection genotypes have different geographical distributions and susceptibilities to infection. In addition, differences among ethnic groups have suggested a genetic contribution in susceptibilities to CP infection. Also, in this study, only the population of the South East of Iran was analyzed which is not representative of the general Iranian CP patients. Therefore, more evidence is required to obtain a conclusion so other SNPs with large enough sample size should be included in the future studies. In summary, our study shows that *IL-28B* gene polymorphisms have an influence on the natural history of chronic periodontitis in a sample of the Iranian population. Single nucleotide polymorphisms *rs12979860* and *rs8099917* can influence the natural history of chronic periodontitis in patients.

## Conclusion

Our findings showed that polymorphisms in *IL-28B* genes (*rs12979860* and *rs8099917*) correlated to the susceptibility to CP infection in our population.

## Abbreviations

CP: Chronic Periodontitis
IL-28B: Interleukin 28B
PCR: Polymerase Chain Reaction
RFLP: Restriction Fragment Length Polymorphism
SNP: Single Nucleotide Polymorphism
